# Improved outcome of critically ill patients treated by the Rapid Response Team outside the intensive care unit

**DOI:** 10.1186/cc10204

**Published:** 2011-06-22

**Authors:** AAFS Georgeto, MT Tanita, PS Taguti, PS Pariz, D Kamiji, MF Sacon, KP Araújo, LTQ Cardoso, CMC Grion

**Affiliations:** 1 Hospital Universitário de Londrina, Universidade Estadual de Londrina, Londrina - PR, Brazil

## Introduction

Due to the limited number of intensive care unit (ICU) beds in Brazilian public hospitals, many critically ill patients are treated in hospital wards while waiting to be transferred to the ICU. Care for these patients is provided by ward staff, while waiting for ICU bed availability. These healthcare providers are not trained in critical care and are not as experienced in caring for ICU patients. In the Londrina University Hospital, the Rapid Response Team (RRT) staff is composed of intensivist healthcare providers who help to deliver specialized care to critically ill patients in general hospital wards.

## Objective

To compare clinical outcomes of critically ill patients treated in general hospital wards in two periods of time, before and after the implementation of a RRT.

## Methods

A prospective longitudinal study developed in two periods: from January to December 2005 before RRT implementation and from January to December 2010 after the RRT is already performing outreach care for critically ill patients. Patients entered the study on the first day an ICU bed was requested and were followed until ICU admission, death or the request for ICU was cancelled due to clinical improvement. The chi-square test was used for statistical analyses.

## Results

We analyzed 699 patients in the first period of 2005 and 889 in the second period of 2010. There was no difference in mortality of these patients comparing the two study periods. We observed an increase in the proportion of patients who presented clinical improvement and had their ICU bed request cancelled in the year 2010 compared with the year 2005 (28.57% vs. 19.03%, *P *< 0.001). There was a decrease in the proportion of patients admitted to the ICU after waiting for bed availability in the second period (45.67 vs. 59.80%, *P *< 0.001) compared with the first period. We also observed the inclusion of end-of-life discussions during routine rounds in these patients outside the ICU and decisions to withhold or withdraw treatment were the reason to cancel an ICU bed request in 34 (3.82%) patients in the year 2010. See Table 1.

**Table 1:** Number of patients according to clinical outcome

	2005		2010	
	
	*n*	%	*n*	%
Patient transfer to another institution*	24	3.43	5	0.56
Death**	124	17.74	190	21.37
Clinical improvement*	133	19.03	254	28.57
ICU admission*	418	59.80	406	45.67
Withhold/withdraw treatment*	0	0	34	3.82
Total	699	100.00	889	100.00

Number of patients according to clinical outcome in Londrina University Hospital, Londrina, Paraná State, Brazil, January to December 2005 and 2010. *P = 0.08. **P < 0.001.

## Conclusion

We observed improvement in clinical outcome of critically ill patients after the implementation of outreach intensive care support delivered by a RRT in a teaching hospital. This effect apparently decreased the need for ICU beds, since more patients improved before an ICU bed was available. We also observed the inclusion of end-of-life discussions in the routine care of these patients.

